# Deep learning architectures for influenza dynamics and treatment optimization: a comprehensive review

**DOI:** 10.3389/frai.2025.1521886

**Published:** 2025-05-27

**Authors:** Adane Adugna, Desalegn Abebaw, Abtie Abebaw, Mohammed Jemal

**Affiliations:** ^1^Medical Laboratory Sciences, College of Health Sciences, Debre Markos University, Debre Markos, Ethiopia; ^2^Department of Biomedical Sciences, School of Medicine, College of Health Sciences, Debre Markos University, Debre Markos, Ethiopia

**Keywords:** deep learning architectures, deep learning models, influenza dynamics, influenza prediction, treatment optimization

## Abstract

As a major worldwide health concern, influenza still requires precise modeling of flu dynamics and efficient treatment approaches. Deep learning architectures are increasingly being applied to address the complexities of influenza dynamics and treatment optimization, which remain critical global health challenges. This review explores the utilization of deep learning methods, such as Long Short-Term Memory (LSTM) networks, Convolutional Neural Networks (CNNs), Generative Adversarial Networks (GANs), transformer architectures, and large language models (LLMs), in modeling influenza virus behavior and enhancing therapeutic strategies. The dynamic nature of influenza viruses, characterized by rapid mutation rates and the emergence of new strains, complicates the development of effective treatments and vaccines. In other words, the discovery of effective treatments and vaccines is severely hampered by the dynamic character of flu viruses, their fast rates of mutation, and the appearance of novel strains. Traditional epidemiological models often fall short due to their reliance on manual data interpretation and limited capacity to analyze large datasets. In contrast, deep learning offers a more automated and objective approach, capable of uncovering intricate patterns within extensive flu-related data, including genetic sequences and patient records. The application of deep learning to comprehend flu dynamics and improve treatment strategies is examined in this review paper. Moreover, this paper discussed relevant research findings, and future directions in leveraging deep learning for improved understanding and management of influenza outbreaks, ultimately aiming for more personalized treatment regimens and enhanced public health responses.

## Introduction

Influenza remains a worldwide health problem, causing seasonal epidemics and occasional pandemics. Influenza viruses result in significant morbidity and mortality (Harrington et al., 2021). Every year, there are about a billion cases of seasonal influenza, with 3–5 million of those cases resulting in serious disease and causes 290,000–650,000 respiratory deaths. In underdeveloped nations, lower respiratory tract infections caused by influenza account for 99% of mortality in children under the age of five (WHO, 2023).

Influenza viruses exist in four different varieties, A, B, C, and D. Seasonal illness outbreaks are brought on by the spread of the influenza A and B viruses (CDC, 2023). Influenza viruses are further divided into subtypes based on the patterns of the virus’s surface proteins. The influenza viruses of subtypes A (H1N1) and A (H3N2) are currently circulating in humans. Since A (H1N1) caused the pandemic, it is often written as A (H1N1) pdm09. The patterns of the virus’s effect, dissemination, and transmission within a population are referred to as influenza virus dynamics (Wille and Holmes, 2020). Comprehending these processes is essential to formulating efficacious tactics to regulate and alleviate the consequences of influenza epidemics. Influenza virus continuously evolves to escape human adaptive immunity and generates seasonal epidemics (Lou and Liang, 2024).

On the other hand, influenza viruses frequently mutate, resulting in antigenic shift (significant alterations brought about by reassortment) or antigenic drift (minor variations in surface proteins). These changes may have an impact on vaccination efficacy and may also be a factor in recurrent pandemics (Nabakooza and Galiwango, 2022).

Conventional approaches for influenza dynamics and treatment optimization have a limitation in that they frequently rely on subjective and time-consuming manual interpretation of medical imaging data. Delays in diagnosis and treatment may result from this, and there may be discrepancies in evaluations made by various medical specialists (Naserpor and Niakan Kalhori, 2019).

Furthermore, a lot of patient data may be difficult for traditional approaches to efficiently evaluate to find intricate correlations or patterns that could guide therapy choices. This restriction may make it more difficult to tailor care according to the unique needs of each patient and the course of their illness (Lou and Liang, 2024). Additionally, the quantity of information found in patient records and medical pictures may not be completely utilized by conventional approaches, which could result in missed possibilities for better treatment optimization (Sansone et al., 2022).

Deep learning, however, presents novel opportunities for examining sophisticated flu-related data and advancing our knowledge of the infection (Su et al., 2019). Moreover, deep learning can overcome these shortcomings of traditional methods by providing a more automated and objective means of assessing patient records and medical imaging data (Ahmed et al., 2023). Comprehending the dynamics of influenza is essential for efficient prevention and treatment (Amirahmadi et al., 2023; Liu et al., 2024). Compared to conventional epidemiological models, deep learning provides an alternative method that enables the capture of complex patterns and temporal correlations (Serghiou and Rough, 2023).

Modeling the dynamics of influenza epidemics and optimizing influenza virus treatment approaches are two applications of deep learning (Du et al., 2020). Deep learning models can offer insights into the spread of the virus and forecast its future trajectory by evaluating massive datasets that include data on flu transmission rates, environmental factors, and patient characteristics (Wanduku et al., 2020; Aiken and Nguyen, 2021).

Through the simulation of various situations and evaluation of their possible results, these models can assist in the identification of the best treatment approaches. Additionally, they can help identify those who are most likely to experience serious flu-related complications and, based on these assessments, suggest individualized treatment regimens (Borkenhagen et al., 2021).

Deep learning can also be used to examine the potential effects of various interventions on the virus’s ability to propagate, such as vaccination drives and antiviral medications. This makes it possible to make better-informed decisions on the implementation of public health measures during influenza outbreaks (Nic-May and Avila-Vales, 2020).

The use of deep learning in predicting the efficacy of influenza virus treatments has grown. Deep learning algorithms are capable of offering important insights into the possible effectiveness of various treatment modalities for influenza virus infections by sifting through massive datasets and finding patterns within them (Aiken and Nguyen, 2021). This strategy could significantly advance our knowledge of the possible effects of specific treatments on patient outcomes, which could ultimately result in more individualized and efficacious therapeutic interventions for influenza virus-affected patients (Adam and Rampášek, 2020). Hence, this review will summarize the application of various deep learning architectures for the prediction of influenza dynamics and treatment optimization.

## Flu dynamics modeling

### Traditional epidemiological models used for flu dynamics

Traditional epidemiological models used for flu dynamics include the SIR (a susceptible-infectious-recovered) model, which splits the population into three compartments-susceptible individuals, infectious individuals, and recovered or immune individuals one of the classic epidemiological models used for flu dynamics (Osthus et al., 2017). To comprehend the disease’s spread, this model monitors changes in the population within each compartment over time.

Furthermore, extending the susceptible-exposed-infected-removed (SEIR) model is an expansion of the SIR model that takes into account the virus’s incubation time by including an “exposed” compartment for infected but non-contagious individuals (Bjørnstad et al., 2020). Additionally, compartmental models can include demographic variables like age and risk groups and divide the population into numerous compartments based on varying stages of infection (Levy et al., 2017).

Transmission dynamics models incorporate characteristics such as pathogen shedding rates and individual contact rates to help explain the spread of influenza within a population. Finally, to explain how the flu spreads across different regions, spatial epidemiological models take into account geographic characteristics in addition to classic epidemiological variables (Goeyvaerts et al., 2015).

Moreover, geographical considerations are taken into account in addition to standard epidemiological variables in spatial epidemiological models, which help explain how the flu spreads across different locations (Ganesan and Subramani, 2021; He et al., 2015).

Conventional epidemiological models facilitate the understanding of flu dynamics by simulating different scenarios for researchers and public health officials. These models also help predict future outbreaks, aid in the effective planning of public health responses, and assess approaches to intervention such as vaccination campaigns or social distancing measures. However, the intricacies of the real world are difficult to capture in these traditional models (Park et al., 2023).

For flu dynamics, deep learning approaches offer an alternative to conventional epidemiological models. Artificial neural networks are used by deep learning techniques to handle large volumes of unstructured data, including genetic sequences or medical imaging (Zan et al., 2022). In contrast to the compartmental models’ dependence on preset compartments and parameters, these approaches offer a more flexible and data-driven method that has demonstrated promise in discovering patterns and forecasting disease outcomes (Shah and Palomar, 2024).

In other words, for flu modeling, deep learning architectures using recurrent neural networks (RNNs) and convolutional neural networks (CNNs) offer scalability and flexibility. Deep learning architectures, including recurrent neural networks (RNNs) and convolutional neural networks (CNNs), provide flexibility and scalability for flu modeling (Sherstinsky, 2020) (Table 1). The table below encapsulates the essential elements of the review paper, focusing on the capabilities of various deep-learning architectures in understanding influenza dynamics and optimizing treatment strategies while contrasting them with traditional methods.

**Table 1:** Traditional vs. deep-learning approaches in influenza dynamics and treatment optimization.

Criteria	Traditional approaches	Deep-learning approaches	References
Data handling	Relies on manual interpretation and limited data analysis capabilities.	Automates analysis of large datasets, revealing intricate patterns.	Sarker (2021)
Modeling flexibility	Fixed compartmental models with predefined parameters.	Data-driven models that adapt based on input data characteristics.	Yu (2022)
Predictive accuracy	Often struggles with complex correlations and temporal dynamics.	Capable of capturing complex temporal patterns, improving forecasting accuracy.	Lin and Huang (2020)
Personalization of treatment	Limited ability to tailor treatments based on individual patient data.	Can analyze patient-specific data to optimize treatment regimens effectively.	Huang et al. (2022)

## Deep learning architectures and their applications for flu dynamics

### Data governance: sources, collection, preprocessing, and quality control

Accurate and appropriate utilization of multi-source, heterogeneous data is crucial for training and validating deep learning models in influenza research. Rigorous scrutiny of data sources, collection timeframes, preprocessing techniques, and quality control measures is essential to ensure the validity of findings (Yang and Li, 2023). These considerations include diverse data sources such as influenza-like illness (ILI) cases, virological surveillance, climate and demographic information, search engine data, and social media data. Integrating public health surveillance data, electronic health records (EHR), internet search trends, and social media activity can also be highly beneficial. Specifying a precise timeframe for data collection is vital; for example, one study gathered data from week 26 of 2012 to week 25 of 2019. Robust data preprocessing is necessary to manage heterogeneous data, encompassing alignment of data with time labels, normalization of values, and correlation and weight analysis to select optimal datasets while avoiding collinearity. Establishing the reliability of data sources is paramount, and resolving data quality and consistency issues is essential for enhancing model reliability.

### Model selection criteria

Model selection is a critical part of building efficient deep learning architectures for flu dynamics and treatment optimizations, where the optimal model is chosen based on specific criteria to achieve peak performance. In this context, models are used for forecasting influenza outbreaks, characterizing antigenic drift, and optimizing treatment options (NEPTUNE.AI, 2025).

Key selection factors include the Akaike Information Criterion (AIC) and Bayesian Information Criterion (BIC), which assess model fit while penalizing complexity; cross-validation, which evaluates model generalizability; Mean Absolute Error (MAE) and Mean Squared Error (MSE), which measure prediction accuracy; computational complexity and resource constraints, which balance performance with efficiency; and interpretability, which is essential for informed decision-making in medical applications (Shah and Palomar, 2024).

Advanced deep learning models, such as Long Short-Term Memory (LSTM) networks for time-series forecasting, Convolutional Neural Networks (CNNs) for image processing, Generative Adversarial Networks (GANs) for generating realistic data, Recurrent Neural Networks (RNNs), and Adaptive Boosting (AdaBoost) and ensemble techniques for enhanced predictive accuracy, are commonly employed (Durr et al., 2023).

### Long-short-term memory (LSTM) for influenza dynamics and treatment optimization

An important development in flu infection rate prediction is the use of LSTM-based deep neural networks trained on historical Influenza-Like-Illness (ILI), climate, and demographic data (Tsan and Chen, 2022). The potential of the model to optimize flu treatment techniques is demonstrated by its ability to anticipate seasonal fluctuations in short-term flu infection rates by taking into account important elements including temperature, precipitation, local wind speed, population size, vaccination rate, and vaccination efficacy (Nic-May and Avila-Vales, 2020). Notably, the discovery that temperature is the best indicator of ILI rates highlights how crucial climatic data are to comprehending the mechanics of flu transmission (Yang et al., 2018).

The LSTM model’s superior performance over other algorithms at the +1 week prediction point demonstrates how well it can capture intricate temporal patterns and more accurately forecast future flu trends (Zhao et al., 2023). This study demonstrates the application of deep learning architectures to maximize targeted flu prevention and treatment tactics and inform public health activities.

LSTM-based recurrent neural networks provide effective short-term flu forecasting. Flu time series prediction can benefit from the superior sequential data handling capabilities of LSTM networks. LSTMs have been employed by researchers to forecast influenza activity based on environmental conditions and previous data (Amendolara et al., 2023). Recurrent neural networks of the LSTM type have been applied extensively to time series prediction applications. LSTM models can be trained with surveillance data, meteorological data, and social media data from sites like Twitter to forecast the spread of influenza-like illnesses (Athanasiou and Fragkozidis, 2023).

The capacity of LSTM-based prediction models to identify long-term patterns and dependencies in sequential data is a benefit. This makes them ideal for catching intricate connections between many variables, like the number of flu cases, the state of the weather, and conversations on social media. Compared to standard epidemiological models, which could have trouble combining non-traditional data sources, LSTM models may be able to provide more accurate forecasts by utilizing these varied information sources (Li et al., 2024).

Moreover, real-time updates from social media streams and monitoring systems can be integrated with LSTM networks to enable dynamic modifications to the prediction model in response to changing circumstances (Zhu et al., 2019). Multi-step influenza epidemic predictions can also be made using adjusted LSTM models. The LSTM model can be used to forecast future epidemic trends over a longer time horizon by training it on past influenza data, such as weekly or monthly case counts, and then using that training data to produce multi-step forecasts. This is how the procedure could appear (Zhang and Nawata, 2018).

Furthermore, the prior study introduced a novel multi-stage forecasting strategy based on LSTM that incorporates the influence of several external variables into state-of-the-art machine learning models (Livieris and Pintelas, 2022).

In LSTM models, the selection of parameters such as time step size and the number of hidden layer units plays a significant role in model performance for predicting flu dynamics and optimizing treatment strategies. The LSTM network is designed to learn long-term dependencies in sequential data, making it an ideal option for time-series prediction, including influenza incidence prediction (Zhu et al., 2022). The inputs, output, and forget gates control the flow of information in the network, allowing the model to selectively remember, update, or forget information at each time step. During training, different hyperparameter settings, such as batch size, number of epochs, and the number of LSTM units, is tested to fine-tune the models for optimal performance (Li et al., 2024). For instance, one experiment attempted LSTM units of sizes 4, 8, 16, 32, and 64, and trained the model for 400, 450, and 500 epochs. The best hyperparameters are empirically tuned to achieve optimal performance (Tsan and Chen, 2022).

A time-series forecasting model is used in the model’s initial stage. In later phases, the spatial proximity of various geographic regions and the situational time lag between the flu incidence and meteorological variables are recorded to correct the inaccuracy produced by the initial forecasting model and enhance the model’s performance even further (Lee et al., 2024).

Drug development and discovery is one application. Molecular structures can be analyzed by deep learning techniques to find possible influenza virus-targeting medication candidates. This may hasten the search for novel antiviral drugs or enhance the efficacy of currently available therapies (Vamathevan et al., 2019).

Personalized medicine is another area of use. Deep learning models can assist in identifying patterns that may indicate which therapies are most successful for particular individuals or subgroups by examining patient data and flu symptoms (Zhang et al., 2019). Moreover, by examining viral mutations and forecasting potential viral evolution, these structures can help in the development of vaccines. This may aid in the creation of more potent vaccinations that offer a wider defense against various influenza virus strains (Thadani and Gurev, 2023).

LSTM models have several applications in individualized treatment planning. First of all, LSTMs use patient data analysis to forecast an individual’s response to particular medications. This allows physicians to more successfully customize treatment regimens based on patient-specific differences (Bica et al., 2021). Additionally, LSTMs reduce adverse effects including age, weight, and genetics while optimizing drug dosage based on patient variables (Wu et al., 2024). Moreover, LSTMs track patient reactions throughout therapy and make necessary modifications when deviations happen. Furthermore, by simulating counterfactual, time-varying, and dynamic treatment options, LSTMs assist physicians in making the optimal decision based on anticipated results (Yilmaz and Büyüktahtakın, 2023).

LSTM has the potential to be used in flu treatment optimization by forecasting the efficacy of various antiviral drugs based on patient data and clinical results. It might be able to create a predictive model that assists medical professionals in selecting the best antiviral therapy for each patient by training an LSTM model on a big dataset of patient records that includes symptoms, test findings, treatment regimens, and results (Zhu et al., 2022).

It is crucial to remember that using LSTMs or any other machine learning technique for medical decision-making necessitates thorough validation and ethical considerations. Before being included in routine medical practice, any application of LSTM for flu treatment optimization would need to go through extensive testing and validation through clinical studies (Aslan, 2024). Overall, even though LSTM has the potential to improve flu treatment strategies through large-scale dataset analysis and predictive modeling, its use in this situation necessitates rigorous thought and medical community confirmation.

### Convolutional neural networks (CNNs) for influenza dynamics and treatment optimization

CNNs were important in ushering in the new era of artificial intelligence and have been at the center of the deep learning revolution. CNNs can analyze complex datasets by converting them into pseudo pictures with little processing for any high-dimensional dataset. They can also identify patterns in photos with scattered pixels (Ersavas et al., 2024). In other words, CNNs are skilled at identifying spatial information in pictures. CNNs have been used in flu studies to evaluate chest X-rays and spot flu-related patterns, assisting with early identification (Montesinos López et al., 2022). CNNs have been used to analyze enormous amounts of data to spot patterns and trends that might not be immediately obvious to human observers (Mustapha et al., 2024). This has allowed CNNs to be applied to a variety of flu dynamics applications, including disease identification, monitoring, and prediction. CNNs are specifically used for flu dynamics in a few ways, such as early diagnosis and treatment assistance by analyzing medical pictures, such as CT or X-rays, to detect diseases (Sarvamangala and Kulkarni, 2022). CNNs may also track the spread and consequences of flu outbreaks in real time by collecting data from many sources, including news articles, social media posts, and medical records. Furthermore, by examining past data on flu cases and pertinent characteristics, CNNs can predict the trajectory and intensity of upcoming flu outbreaks.

In CNNs for flu dynamics and treatment optimization, the optimal selection of key parameters is crucial. The architecture of CNNs primarily consists of convolutional layers, pooling layers, and fully connected layers, with parameters like filter sizes, the number of filters, and stride influencing the model’s capacity for recognizing significant features from input data1. For instance, to forecast the host tropism of influenza A viruses, a CNN model (Flu-CNN) utilized six convolutional layers and three fully connected layers, with ReLU and Pooling in the convolutional layers and ReLU and Dropout in the fully connected layers. The training epoch and batch size are crucial parameters, with cross-entropy serving as the loss function. The choice of activation functions, such as ReLU, addresses the vanishing gradient problem and reduces the dependency between neurons. Furthermore, techniques like dropout can prevent overfitting of the network, enhancing its performance on new samples.

To infer influenza antigenic variations, a CNN model has been developed (IAV-CNN) (Yin et al., 2022). It efficiently examines the relationships between the HA1 sequence’s amino acid locations. Also, CNN does not include any amino acid embeddings because it only concentrates on the physicochemical characteristics that are essential for the antigenicity of influenza viruses (Jorquera et al., 2019). CNN can capture the interactions between different amino acid positions in the HA1 sequence and analyze how point mutations affect antigenic variation as a whole. Retrospective testing and 5-fold cross-validation have demonstrated that CNN outperforms its competitors in the prediction of antigenic variations. CNN has also helped determine the main antigenic clusters for A/H3N2 (Meng et al., 2024).

In the earlier work, an ensemble CNN with weights for predicting influenza virulence, a virus known as VirPreNet that utilizes all eight segments, was proposed. The core component of VirPreNet is the ensemble CNN, which is built as the basis model using the influenza dataset of each segment after the influenza strains have been divided and embedded. VirPreNet delivers state-of-the-art performance, according to the experimental results on the gathered influenza dataset. Additionally, this model highlights the significance of the PB2 and HA regions in predicting pathogenicity (Yin et al., 2021).

According to the results of a prior study, the CNN model can considerably reduce the amount of time needed to identify influenza virus-induced cytopathic effects (CPE). The performance of this model to recognize CPE effects was 99.75% (Wang et al., 2020).

In the study of influenza dynamics, CNNs have been used to forecast and assess the virus’s propagation. It is also useful for predicting the temporal dynamics of infectious diseases such as influenza. They have also been effectively applied to time-series data analysis (Huang et al., 2022).

CNNs have been applied to influenza dynamics, for example, by analyzing spatiotemporal patterns in the transmission of the disease. By using past data from flu epidemics to train a CNN, researchers can find patterns and trends in the way the virus spreads over time across various geographic regions. This can assist in forecasting upcoming epidemics and guiding public health initiatives (Cai et al., 2019). CNN are essential for optimizing flu therapy because they can improve diagnosis and individualized care by improving antigenic variant prediction. Their uses include precision therapy and personalized medicine in addition to flu treatment (Vaz and Balaji, 2021).

CNNs have the potential to be applied in multiple ways to optimize flu treatment. Analyzing medical imaging data, such as chest X-rays or CT scans, to assist in the diagnosis and monitoring of problems connected to influenza infections is one possible use of CNNs for flu therapy optimization (Li et al., 2023). It might be possible to create a model that helps medical professionals recognize and treat flu-related problems more successfully by training a CNN on a sizable collection of medical images from patients with these difficulties (Serafim et al., 2021).

Additionally, CNNs could be used to forecast the effectiveness of antiviral medications against certain strains of the flu virus by studying their molecular structures. Through the training of a CNN using molecular data and drug response profiles, scientists could potentially discover novel compounds or enhance current treatments to more effectively combat influenza (Singh and Singh, 2023). Using information about a patient’s genetic composition, medical history, and other pertinent factors, CNNs can also be used to predict how a particular patient would react to various flu treatments. This individualized approach may result in more successful treatment regimens created especially for each patient (Ali et al., 2023).

It is crucial to remember that whereas CNNs have intriguing opportunities for enhancing flu treatment plans via image analysis and medication creation, their use in healthcare necessitates strict validation through clinical trials and regulatory approval procedures. Additionally, in using deep learning models in medical settings, ethical concerns about patient privacy and informed permission must always take precedence (Sreeraman et al., 2023).

Large quantities of patient data and medical records can also be analyzed by CNNs to find patterns and connections that might not be immediately obvious to human observers. CNNs can aid in the optimal selection of antiviral medications or other therapies by utilizing this capability and taking into account each patient’s particular characteristics and the course of their condition (Derry et al., 2023).

### Generative adversarial networks (GANs) for influenza dynamics and treatment optimization

GANs and their variants have become transformative tools in medical imaging, addressing challenges like data scarcity, modality translation, and diagnostic accuracy enhancement. These models excel in tasks ranging from synthetic data generation to complex predictive analytics, enabling advancements in clinical decision-making (Heng et al., 2024). In other words, GANs offer significant opportunities to address critical gaps in influenza research; specifically, in data augmentation, GANs could synthesize viral sequences or multi-modal medical imaging (e.g., lung CT scans) to alleviate data scarcity in low-resource settings, mirroring their successful application in augmenting chest X-rays for pneumonia detection (Lan et al., 2020).

Furthermore, GANs could transform molecular design by employing adversarial frameworks to optimize antiviral medications such as oseltamivir, akin to how machine learning models predict antigenic properties of influenza viruses, potentially improving vaccine strain selection (Murad and Ali, 2023). In addition, GANs hold promise for immune response simulation, where they might model cytokine storm dynamics or immune cell interactions, perhaps leading to the identification of biomarkers for severe influenza outcomes (Berman et al., 2023).

Flu dynamics and treatment optimization are two areas where GANs are being applied for a variety of reasons. They can produce synthetic flu data that is useful for running simulations and training predictive models since it closely mimics real-world dynamics. Better forecasting accuracy can also be achieved by using GAN-generated data to enhance predictive models and offer a more varied dataset for study (Lan et al., 2020).

With the generation of synthetic data that closely mirrors real-world patterns, GANs can help resolve data deficiency issues common in healthcare, particularly in influenza studies where timely and diverse data is often scarce (Murad and Ali, 2023). This capability facilitates better training and testing of models, enhancing the predictability of disease spread and severity forecasts. Additionally, GANs can simulate diverse scenarios, allowing scientists to explore various treatment strategies and identify optimal interventions based on simulated outcomes (Arora and Arora, 2022). This approach not only aids in developing improved treatment approaches but also supports public health decision-making by providing insights into potential disease trajectories under different conditions. In the study of flu dynamics, GANs can be used in addition to CNNs. Synthetic flu data can be produced by GANs, which improves analysis and comprehension of flu dynamics (Paladugu et al., 2023). GANs support a more thorough approach to disease detection, monitoring, prediction, and vaccine development in the context of influenza outbreaks by giving CNNs access to extra data for analysis. Artificial flu data can be produced by GANs, supplementing small datasets used for model training. Additionally, they make data augmentation for reliable flu dynamics modeling easier (Mamo et al., 2024).

By modeling various scenarios based on generated data, GANs also help optimize treatment methods by exploring the possible effects of interventions like vaccination campaigns or antiviral medications. Lastly, by seeing odd patterns in flu data that might point to new strains or unique transmission patterns, GANs could help with anomaly detection (Abbasi et al., 2022).

GANs, a powerful class of neural networks with two neural networks, such as a discriminator and a generator are utilized for unsupervised learning (Durr et al., 2023). To provide believable data that mimics real data, the generator generates false data, such as photographs, which are subsequently trained on the discriminator (Kazeminia et al., 2020). This fabricated data is generated by GANs using adversarial training, which qualifies them for flu treatment optimization. The learning rate, batch size, and choice of activation functions are all critical parameters in GANs, significantly influencing the quality of the generated data. Effective training also requires careful tuning of the loss functions for both the generator and discriminator. This tuning is essential to ensure stable convergence and to prevent mode collapse, a scenario where the generator produces only a limited set of samples (Kdnuggets, 2025).

Moreover, style transfer, data enrichment, and creation of images are only a few of the uses for GANs. There are numerous ways that GANs can be used to provide personalized flu treatment (Putin et al., 2018). First, by creating the molecular structures of possible antiviral medications and refining their chemical characteristics to find attractive drug candidates, GANs can be utilized for drug discovery and optimization. Furthermore, by evaluating an individual’s immune response data and customizing vaccine components to meet their unique requirements, GANs can help build individualized flu shots, increasing efficacy and minimizing side effects (Lin et al., 2020).

GANs are also useful for customizing treatment plans by forecasting therapy responses based on patient data. Additionally, based on patient factors like age, weight, genetics, etc., GANs can adjust medicine dosage to maximize therapeutic benefits while reducing negative effects (Gan et al., 2023).

By training the generator network on currently available therapies and using the discriminator network to evaluate each therapy’s efficacy according to predetermined criteria, GANs can produce synthetic data that represents different flu treatment alternatives, such as antiviral medications and therapeutic antibodies (Xie et al., 2022).

Furthermore, the discriminator network inside the GAN framework can assess the efficacy of created therapies in fighting influenza by taking into account parameters like molecular structure and possible adverse effects. Furthermore, when developing and accessing flu therapies, GANs may be configured to take into account the specific traits of each patient as well as genetic variability and this allows for the recommendation of individualized treatment plans that are best suited to each patient’s distinct biological composition (Ahmad et al., 2024).

However, it is important to remember that even though GANs have the potential to improve flu treatment selection, rigorous validation through real-world data and clinical trials will be necessary before any AI-driven treatment choices are put into practice because of issues with guaranteeing the security and dependability of AI-generated suggestions in medical settings (Ghebrehiwet et al., 2024). Moreover, generating high-quality synthetic data, especially for complex data types such as discrete categorical data or text, can be challenging (Impetus, 2024). GANs may require significant computational resources and extensive training to achieve optimal results. Furthermore, it is critical that the synthetic data accurately reflect the statistical characteristics and patterns of the real data; otherwise, the generated data may be unsuitable for downstream tasks such as flu dynamics modeling and treatment optimization (Arora and Arora, 2022) (Table 2).

**Table 2:** Deep-learning architectures for influenza dynamics.

Deep-learning architecture	Applications	Key features	References
Long short-term memory (LSTM)	Predicting seasonal fluctuations in influenza infection rates Optimizing treatment techniques Analyzes time series data from various sources, and identifies complex patterns. Personalized medicine	Handles sequential data effectively Captures intricate temporal patterns Adapts to real-time data inputs	Shah and Palomar (2024), Amendolara et al. (2023), and Yang and Li (2023)
Convolutional neural networks (CNNs)	Analyzing medical imaging data Extracting features relevant to flu dynamics Identifying patterns in flu-related datasets	Excellent for image recognition tasks Can process spatial hierarchies in data	Yin et al. (2021), Singh et al. (2024), Yin et al. (2022), and Xia et al. (2021)
Generative adversarial networks (GANs)	Simulating potential viral mutations Enhancing vaccine development	Comprises two networks (generator and discriminator) that compete against each other Generates new data samples based on training data Useful for modeling complex distributions Useful for generating synthetic data to augment training datasets or simulate potential flu outbreaks.	Shah and Palomar (2024), Berman et al. (2023), Swanepoel (2023), and Marquioni and de Aguiar (2021)

The table below summarizes the various deep learning architectures discussed in the review paper along with their applications and key features.

## Other deep learning architectures for flu dynamics and treatment optimization

### Transformer architectures

Transformers are powerful deep learning models that leverage self-attention mechanisms to efficiently process and generate sequential data. This technique allows them to quickly understand relationships, even between distant elements within a sequence (Geeksforgeeks, 2025). Their key advantage lies in their ability to model long-range dependencies and contextual information, making them exceptionally well-suited for tasks like language modeling, machine translation, and text generation (Singh and Raman, 2024). In these areas, transformers have driven significant advancements due to their superior performance and ability to capture nuanced relationships within data. In addition, the transformer model employs an encoder-decoder architecture, where both components consist of layered self-attention mechanisms and feed-forward neural networks (Kdnuggets, 2025; Denecke et al., 2024). This design enables parallel processing of input data, making it highly efficient and effective for sequential tasks (Li et al., 2021).

The encoder processes input sequences, creating meaningful representations that the decoder then uses to generate outputs, taking into account both the encoded information and previously predicted tokens. Working in tandem, the encoder and decoder transform the input into a desired output, such as translating languages or generating responses to queries (Machinelearningmastery, 2025).

Transformer architectures have become increasingly popular in healthcare applications (Anwar et al., 2025). Transformer models represent an innovative resource within healthcare and epidemiology, particularly in understanding influenza dynamics and optimizing treatment. Capable of employing self-attention mechanisms to process disparate data types, such as past flu incidence rates, genomic data, social media trends, and patient medical records, transformer models enhance predictive efficiency in forecasting outbreaks and resource utilization (Yang et al., 2023). Their ability to interpret temporal and spatial trends is useful for modeling disease spread, identifying emergent strains via genomic data, and tailoring public health interventions based on factors like mobility and vaccine coverage (Nerella et al., 2024).

At a clinical level, transformers streamline treatment regimens by incorporating patient history, biomarkers, and treatment responses to prescribe antiviral treatments on an individual basis (Nerella et al., 2023). These models also expedite drug discovery by predicting molecular interactions and prospecting potential antiviral medicines. Beyond analytics, transformers improve public health messaging through sentiment analysis of vaccination perceptions and drive real-time surveillance platforms that combine data from labs and hospitals to aid in early threat detection. These applications highlight their value in bridging genomic surveillance to resource planning during flu outbreaks (Cho et al., 2024).

Moreover, White-box Time Series Transformer (WhiteTST) framework achieves cutting-edge accuracy on influenza-like illness (ILI) datasets, thanks to rigorous experimental validation and the use of white-box transformers such as CRATE. The self-attention maps in WhiteTST offer valuable insights into its explainability. Additionally, WhiteTST employs basic white-box models for ILI forecasting that demonstrate both high accuracy and strong interpretability (Shen et al., 2024; Tian et al., 2023). In other words, the transformer-based model leverages the potential of the Transformer architecture to enhance prediction capacity. This model delivers approximate performance in short-term forecasting and superior performance in long-term forecasting (Li et al., 2021).

### Large language models (LLMs)

LLMs are artificial intelligence models trained on vast amounts of text data to produce human-like outputs. They have been applied to a wide range of tasks in healthcare, from answering medical examination questions to generating clinical reports (Busch et al., 2025). Training foundation models, including large language models, on multiple datasets before application enhances performance. Consequently, a time series model is trained using multiple datasets from diverse domains and viral diseases. Additionally, a pre-trained large language model is utilized and adapted for influenza-like illness (ILI) estimation. Furthermore, LLMs are revolutionizing healthcare by enhancing patient engagement and education through personalized materials, improving clinical decision support by analyzing large datasets and automating administrative tasks, and facilitating communication between patients and clinicians via reliable translations (Omiye and Gui, 2024).

LLMs also significantly contribute to clinical documentation by summarizing extensive patient notes and reports, thereby enhancing accuracy and efficiency. Additionally, they aid in diagnosis, treatment decisions, and medical research by parsing complex clinical information and generating valuable insights (Meng et al., 2024). LLMs support patient care by answering medical questions, simplifying complex information, and empowering patients to participate in their care decisions. Despite these advancements, challenges persist, including limitations in contextual understanding and ethical concerns (Aisera, 2025).

Specifically, the novel LLM4cast architecture encodes input patches through a bidirectional encoder to extract rich embeddings. Subsequently, these encoded patches are passed to a pre-trained TinyLlama for fine-tuning. Finally, the output from TinyLlama is flattened and projected to estimate probable ILI cases (Saeed et al., 2024).

**Figure 1:**
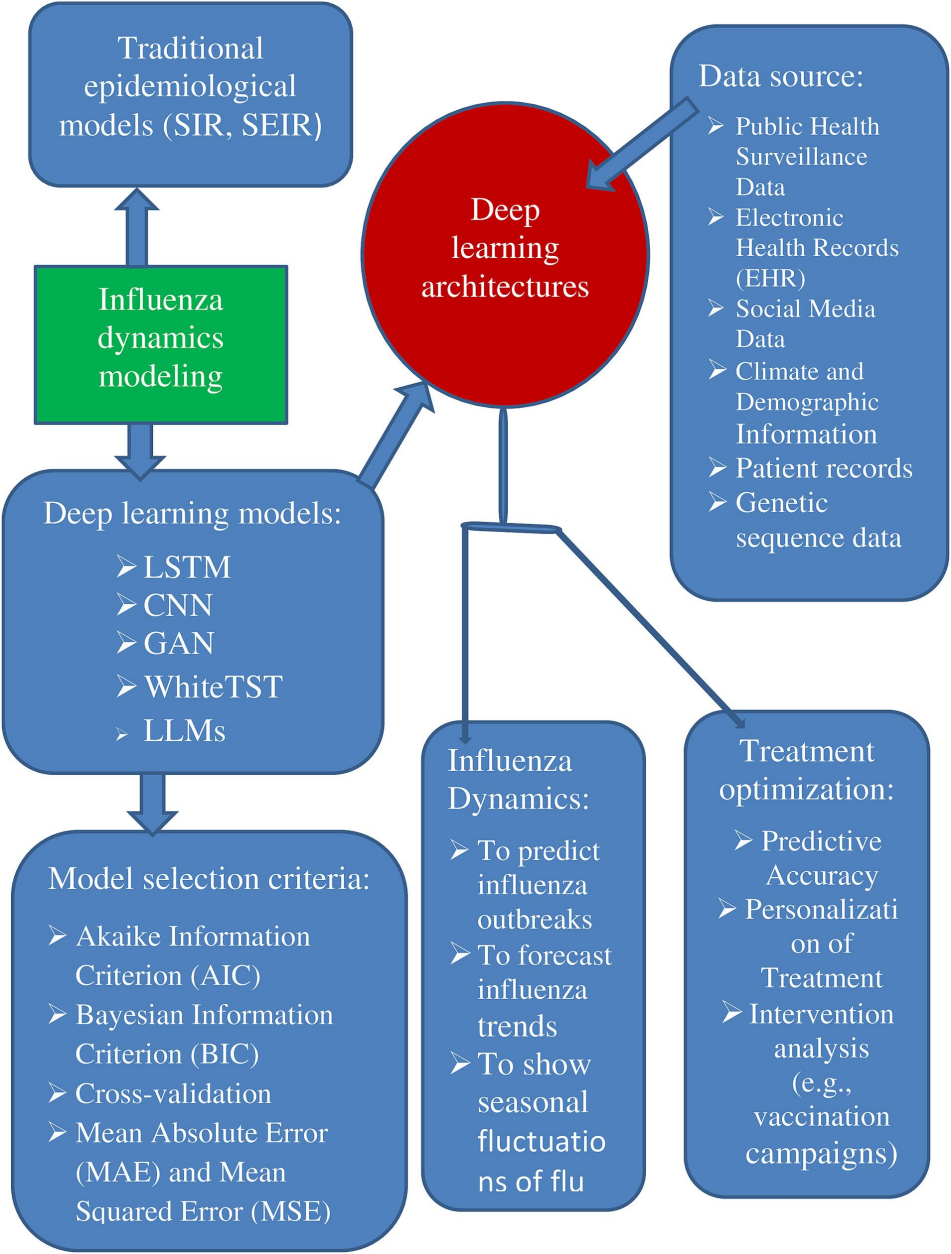
Deep learning applications in influenza dynamics and treatment optimization.

In addition, another LLM model, the spatiotemporal language models (SPLLM), is a large language model or a mathematical model applied to flu dynamics, its potential applications could include analyzing vast amounts of data from flu outbreaks to identify spread patterns and optimize public health responses (Busch et al., 2025). Additionally, it could be used to optimize treatments by leveraging data from within-host models to predict how different interventions affect viral load and immune response (Meng et al., 2024). The SPLLM model might also enable personalized medicine by developing tailored treatment plans based on unique patient data and responses to influenza infections. These applications are speculative and based on the broader potential of LLMs and mathematical models in medicine, as they lack specific details about the SPLLM model (Figure 1) (Denecke et al., 2024).

### Clinical validation of deep learning models in healthcare setting: for influenza prediction and treatment optimization

Clinical validation is crucial for ensuring the reliability and effectiveness of deep learning (DL) models in healthcare, especially in complex fields like influenza dynamics and treatment optimization, bridging the gap between *in-silico* validation and real-world application (Hosny et al., 2022). This entails several significant elements, beginning with establishing clinical benchmarks to ascertain the current standard of care (Wolterskluwer, 2025). Primary validation establishes the DL models’ generalizability on large external datasets through stepwise testing to confirm consistent performance. Functional validation then investigates failure modes, test–retest stability, and accuracy to establish their utility in real-world applications (Chi et al., 2023).

Moreover, end-user testing integrates expert assessments of automated segmentations or predictions in simulated clinical settings to confirm compliance with clinical specifications (Javed et al., 2024). For influenza dynamics and treatment optimization, DL algorithms can enhance the accuracy of real-time prediction through the integration of multiple data modalities from electronic health records, thereby enabling clinical decision-making in emergency departments, whereas DL models such as LSTMs, CNNs, and GANs, integrated with feature engineering methods such as PLE-DT, can predict clinical outcomes from variable-length time series data in clinical settings with high accuracy (Park and Choi, 2021; Zafar et al., 2023).

Furthermore, AI can automate coding and clinical validation processes, increasing efficiency and accuracy in managing a wide range of conditions. However, clinical validation faces limitations including data quality considerations, inconsistency in segmentation patterns among experts, and generalizability across various data collection methods, patient populations, and clinical practices (Bashiri et al., 2024; Prateek and Rathore, 2025). Because in-silico geometric segmentation scores may or may not reflect clinical utility in all instances, functional validation and end-user testing are crucial for identifying the practical implications of DL models in healthcare settings (Helaly et al., 2024; Badawy et al., 2023). Through thorough examination of these aspects and concerns, clinical validation enables effective translation of DL models into effective tools for influenza control and treatment optimization, thereby increasing patient safety and healthcare provision.

## Limitations and challenges of deep learning models in healthcare applications

### Model overfitting, data scarcity, and model interpretability

Deep learning has emerged as a powerful tool for predicting influenza dynamics and treatment optimization. However, several challenges must be addressed to enhance its effectiveness.

Overfitting is a common issue in machine learning where a model becomes too specialized to the training data, capturing not only the underlying patterns but also the noise and random fluctuations, leading to poor performance on new, unseen data (Huang et al., 2018). Overly complex models may perform well during training but poorly during the validation or testing phases of research (Takahashi et al., 2020). This issue is particularly prevalent in complex models like LSTMs, CNNs, and GANs, which can learn too many parameters from the training data, and in cases where the training data is small or noisy, or when models are trained for too long. Overfitting can have significant consequences, especially in critical applications such as medical diagnosis or financial forecasting. For reliable outcomes, strategies to prevent overfitting in deep-learning models are essential for influenza dynamics and treatment optimization (Chaudhary et al., 2018).

To prevent overfitting, techniques like regularization, data augmentation, ensemble methods, cross-validation, and dropout are employed (Salehin and Kang, 2023). In the context of influenza dynamics modeling, these strategies are crucial for developing accurate predictive models (Madaeni et al., 2022). In addition, a diverse and sufficiently large dataset can also reduce overfitting in deep-learning models (Bejani and Ghatee, 2021).

Deep learning models like LSTMs, CNNs, and GANs require specific approaches to mitigate overfitting: LSTMs use dropout and cross-validation, CNNs benefit from data augmentation and regularization, and GANs utilize batch normalization and heterogeneous datasets. Ensuring a large and diverse dataset is also essential for preventing overfitting in these models (Ng et al., 2023).

In addition to model overfitting, deep learning models for influenza dynamics face significant challenges like data scarcity and model interpretability (Choudhary et al., 2022). The scarcity of data is a major issue, as high-quality, timely, and comprehensive information on influenza cases is limited, making it difficult to train and cross-validate models effectively (Hakami, 2024). In other words, deep learning models need high-quality and diverse data, exploring multimodal data fusion, and developing explainable AI methods. Deep learning models require the integration of diverse data types, such as social media and meteorological data for effective predictions, combining these varied data sources while maintaining accuracy poses a significant challenge. Additionally, the need for real-time updates in predictive models to reflect changing epidemiological circumstances is crucial but challenging, as incorporating dynamic modifications based on new data inputs requires sophisticated modeling techniques that are still being developed.

Additionally, the interpretability of complex deep neural networks, such as LSTMs, CNNs, and GANs, poses another challenge because understanding how these models make their decisions is crucial in healthcare applications where transparency and credibility are essential (Alzubaidi et al., 2023).

Furthermore, the black-box nature of these models hinders the interpretability of their outputs, limiting trust and acceptance in clinical practice. To increase the adoption of deep learning models in clinical practice, future research should prioritize the development of interpretable artificial intelligence (XAI) methods (Amendolara et al., 2023; Thakur, 2024). Over-reliance on historical data and a potential inability to adapt to novel situations, such as pandemics lacking historical precedent, are also limitations. Addressing data quality and consistency, particularly in situations with disrupted healthcare systems, is essential for enhancing model reliability (Teng et al., 2022).

Furthermore, incorporating external variables such as weather patterns or social data could improve predictive power. In addition, overcoming these challenges requires innovative strategies, including the use of diverse data sources like search queries and mobility data, and developing methods to enhance model explainability (Bansal et al., 2022).

### Bias, ethical considerations and data privacy

Bias in deep learning models applied to flu dynamics and treatment optimization can arise from various factors, including subjective clinical judgments, heterogeneous diagnostic criteria, and unequal distribution of healthcare resources. These biases can impact model performance, especially when training data reflect existing disparities or when models are not thoroughly validated across diverse populations. For example, models may perform differently in resource-constrained settings or among high-risk groups, potentially leading to misdiagnosis or suboptimal treatment recommendations. Mitigating these biases requires meticulous data curation, robust validation procedures, and collaboration between clinicians and AI systems to create equitable and accurate decision-making tools for flu diagnosis and treatment optimization.

Although deep learning offers potential for personalized medicine, the challenge remains in accurately predicting individual responses to treatments based on complex interactions between genetic, environmental, and clinical factors; thus, ethical considerations regarding data privacy, consent, and the implications of automated decision-making in clinical settings must also be addressed.

Ethical considerations and data privacy are paramount when deploying DL models in healthcare, especially in influenza modeling and treatment optimization. The use of vast amounts of individual-level health data to train DL models raises significant ethical and legal concerns, necessitating adherence to established ethical frameworks that regulate clinical practice and technology design (Panigutti et al., 2021). Integrating key ethical concepts such as privacy, fairness, and explainability throughout the machine learning pipeline is essential. Principles of beneficence and non-maleficence ensure that AI technology benefits patients and minimizes harm through errors, biases, or misuse, while respecting patient autonomy by maintaining transparency and consent in interactions with AI (CDC, 2021).

Concepts of fairness and justice ensure that AI-driven technologies do not create or exacerbate inequalities, but rather promote equitable access to healthcare services. Moreover, fully harnessing the potential of AI in healthcare requires addressing major ethical issues such as informed consent for data usage, safety and transparency, algorithmic fairness and bias mitigation, and data privacy protection. Therefore, experts and practitioners must consider all four core medical ethics principles, autonomy, beneficence, non-maleficence, and justice, in all facets of healthcare before integrating artificial intelligence into the healthcare system. These challenges highlight the need for ongoing research and development in both deep learning methodologies and their application to public health strategies for influenza management.

## Conclusion

The integration of deep learning architectures such as Long Short-Term Memory (LSTM) networks, Convolutional Neural Networks (CNNs), Generative Adversarial Networks (GANs), transformer architectures, and large language models (LLMs) into influenza research represents a paradigm shift in understanding and managing influenza dynamics. Traditional epidemiological models often fall short due to their reliance on manual data interpretation and limited capacity to analyze large datasets. In contrast, deep learning offers a more automated, objective, and scalable approach capable of uncovering intricate patterns within extensive flu-related data, including genetic sequences and patient records. This advancement not only enhances the predictive capabilities regarding influenza outbreaks but also optimizes treatment strategies by personalizing therapeutic regimens based on individual patient data.

The findings underscore that deep learning methodologies can significantly improve the forecasting of influenza trends, the effectiveness of treatment modalities, and the overall public health response to outbreaks. By harnessing vast datasets from various sources, including social media and environmental factors, these models can provide timely insights that are crucial for effective intervention strategies.

### Future directions

The future of influenza research and treatment optimization lies in several promising directions that harness the power of deep learning. Enhanced predictive models will focus on refining LSTM networks and other neural architectures to improve multi-step forecasting capabilities for influenza outbreaks by integrating diverse data sources. In the realm of personalized medicine, developing models that analyze individual patient data will facilitate tailored treatment approaches, predicting responses to specific therapies. Real-time data integration will leverage information from social media and health monitoring systems, allowing for dynamic updates to predictive models and improving their accuracy in response to shifting epidemiological patterns. Moreover, deep learning techniques will play a crucial role in vaccine development by analyzing viral mutations to predict potential strains, thereby enhancing protection against various influenza virus types. In drug discovery, these techniques will expedite the identification of new antiviral candidates through molecular analysis, potentially increasing treatment efficacy. Moreover, the creation of decision-support systems utilizing deep learning insights will empower public health officials to implement timely interventions during influenza outbreaks, ultimately leading to more effective management of this persistent global health challenge.

In addition, future applications of deep learning models to model influenza dynamics will be significantly influenced by public health interventions, vaccination coverage, and socioeconomic determinants. Robust public health interventions, including timely responses and resource allocation guided by deep learning forecasts, are critical, especially in regions with weakened healthcare systems1. Models incorporating vaccination coverage data can provide insights into the effectiveness of vaccination campaigns and enable the optimization of vaccine distribution plans. Socioeconomic determinants that influence both susceptibility to infection and access to healthcare need to be incorporated into such models to enhance their predictive ability and to enable equitable public health responses. Furthermore, the development of explainable AI (XAI) methods will be needed to enhance the acceptability and trustworthiness of deep learning models in clinical practice.

By focusing on these directions, future research can significantly advance our understanding of influenza dynamics and improve treatment optimization strategies, ultimately leading to better public health outcomes.
